# Sepsis—Pathophysiology and Therapeutic Concepts

**DOI:** 10.3389/fmed.2021.628302

**Published:** 2021-05-14

**Authors:** Dominik Jarczak, Stefan Kluge, Axel Nierhaus

**Affiliations:** Department of Intensive Care Medicine, University Medical Center Hamburg-Eppendorf, Hamburg, Germany

**Keywords:** sepsis, septic shock, immune pathology, inflammation, sepsis therapy

## Abstract

Sepsis is a life-threatening condition and a global disease burden. Today, the heterogeneous syndrome is defined as severe organ dysfunction caused by a dysregulated host response to infection, with renewed emphasis on immune pathophysiology. Despite all efforts of experimental and clinical research during the last three decades, the ability to positively influence course and outcome of the syndrome remains limited. Evidence-based therapy still consists of basic causal and supportive measures, while adjuvant interventions such as blood purification or targeted immunotherapy largely remain without proof of effectiveness so far. With this review, we aim to provide an overview of sepsis immune pathophysiology, to update the choice of therapeutic approaches targeting different immunological mechanisms in the course of sepsis and septic shock, and to call for a paradigm shift from the pathogen to the host response as a potentially more promising angle.

## Introduction

Sepsis is a life-threatening clinical condition with extensive physiological and biochemical abnormalities. The Third International Consensus (Sepsis-3) currently defines sepsis as “organ dysfunction caused by a dysregulated host response to infection”, emphasizing for the first time the crucial role of the innate and adaptive immune response in the development of the clinical syndrome (1). Approximately 49 million people are affected by sepsis every year and it is estimated that 11 million deaths are caused by the syndrome, accounting for up to 19.7% of all deaths worldwide (2). Globally, mortality rates seem to be declining on average, however, up to 25% of patients still succumb to sepsis. In septic shock, a subgroup of sepsis characterized by profound circulatory, cellular and metabolic abnormalities, the hospital mortality rate approaches 60% (3).

Comprehensively defining “sepsis” has been subject of constant development and refinement over the last decades. Although our understanding of origin, pathophysiology, and immunological mechanisms of sepsis has made progress during the last three decades, our options of successful and specific therapeutic interventions remain restricted to non-existent. Only timely fluid resuscitation and early administration of broad-spectrum antibiotics have been shown to reduce mortality. A decisive factor is the time of correct diagnosis and the initiation of causal, supportive, and adjunctive measures. This implies that increasing awareness of sepsis and the promotion of quality improvement initiatives in the field of sepsis effectively improve patient survival, together with the development of novel diagnostics and interventions (4).

## Sepsis Pathophysiology

In contrast to an uncomplicated and localized infection, sepsis is a multifaceted disruption of the finely tuned immunological balance of inflammation and anti-inflammation. The upregulation of pro- and anti-inflammatory pathways leads to a system-wide release of cytokines, mediators, and pathogen-related molecules, resulting in activation of coagulation, and complement cascades (5).

**Figure 1:**
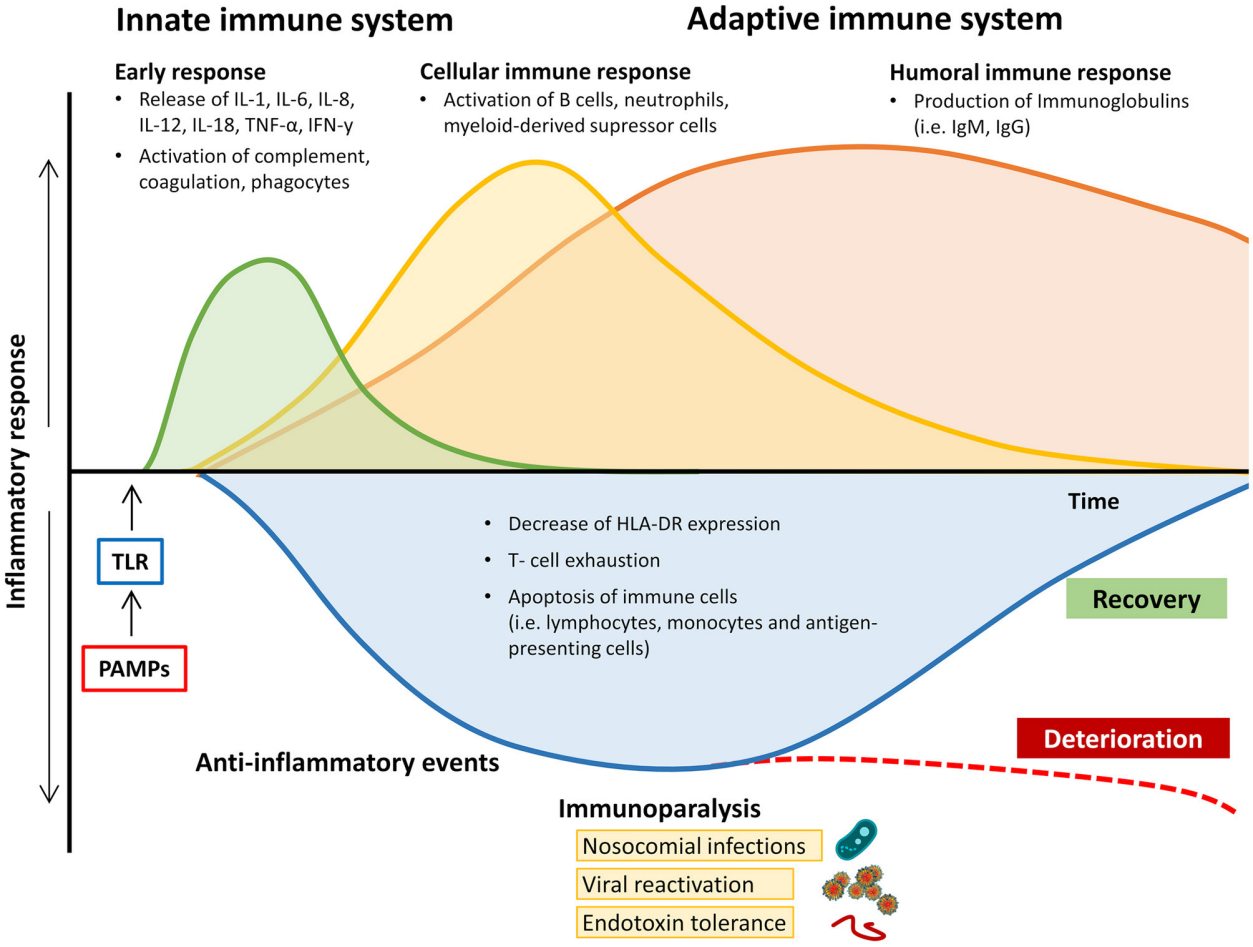
Changes in pro- and anti-inflammatory response of the immune system during the course of sepsis and septic shock. HLA-DR, human leukocyte antigen-D related; IgM/G, immunoglobulin M/G; IL, interleukin; IFN-y, Interferon y; PAMPs, pathogen-associated molecular patterns; TNF-α, tumor necrosis factor alpha; TLR, toll-like receptor.

Recognition of pathogen-derived molecular patterns (PAMPs, e.g., endo- and exotoxins, lipids, or DNA sequences) or endogenous host-derived danger signals (damage-associated molecular patterns; DAMPs) is the starting signal. These molecules activate specific receptors (toll-like receptors, TLR) on the surface of antigen-presenting cells (APCs) and monocytes, thereby initiating the clinical syndrome of sepsis via transcription of genes involved in inflammation, cell metabolism, and adaptive immunity (6). While both pro-inflammatory and anti-inflammatory pathways are upregulated, the resulting inflammation leads to progressive tissue damage, finally causing multi-organ dysfunction. In many patients the concomitant immunosuppression, which is caused by downregulation of activating cell surface molecules, increased apoptosis of immune cells, and T cell exhaustion, leads to “immunoparalysis” in the later stages of the disease course and makes affected patients susceptible to nosocomial infections, opportunistic pathogens, and viral reactivation (Figure 1) (7, 8). Binding of PAMPs and DAMPs to TLRs on APCs and monocytes results in signal transduction, causing translocation of nuclear factor-kappa-light-chain-enhancer of activated B cells (NF-κB) into the cell nucleus. This leads to the expression of “early activation genes,” including various pro-inflammatory interleukins (IL), e.g., IL-1, IL-12, IL-18, tumor necrosis factor alpha (TNF-α), and interferons (IFNs). These subsequently cause the activation of further cytokines (e.g., IFN-y, IL-6, IL-8), complement and coagulation pathways, and, by negative feedback, downregulation of components of the adaptive immune system (9). These processes can be observed during the early stages of the septic disease by an increase in both pro-inflammatory and anti-inflammatory cytokines (8, 10, 11). The net effect on the immunological phenotype (hypo- vs. hyper-responsiveness) remains highly individualized and causes considerable diagnostic difficulties.

As part of the innate immune system, neutrophils form a significant part of the first line of defense against pathogens. Severe bacterial infections induce the release of both mature and immature forms of neutrophils from the bone marrow through emergency granulocyte maturation. When activated via PAMPs or DAMPs, immature neutrophils show reduced phagocytosis and oxidative burst capacity (13–15).

Clinical deterioration is often associated with the detection of elevated levels of these cells, which is in turn associated with increased spontaneous production and release of neutrophil extracellular traps (NETs) (16, 17). NETs are diffuse extracellular structures, consisting of decondensed chromatin with granular and nuclear proteins that have the potential to immobilize a wide range of pathogens. These include Gram-positive and Gram-negative bacteria, viruses, yeasts, but also protozoa and parasites that cannot be regularly phagocytized due to their size (18–20). The release of NETs is known to be triggered by cytokines and chemokines, but also by platelet agonists (i.e., thrombin, ADP, collagen, arachidonic acid) and antibodies (21). Increased occurrence of NETs either due to overproduction or to insufficient degradation has been shown to be associated with hypercoagulation and endothelial damage (17, 22, 23).

### Sepsis-Induced Coagulopathy (and the Role of Endothelium in Sepsis)

Sepsis is frequently associated with coagulopathy, which is an important complication and contributes to the development of organ dysfunction. A recently published analysis of 1895 patients from Japan showed that 29% of critically ill patients with sepsis were diagnosed with sepsis-induced coagulopathy, which is synonymous with disseminated intravascular coagulation (DIC) (24). DIC was defined by the International Society on Thrombosis and Haemostasis (ISTH) as “an acquired syndrome characterized by the intravascular activation of coagulation with loss of localization arising from different causes. It can originate from and cause damage to the microvasculature, which, if sufficiently severe, can produce organ dysfunction” (25, 26). Sepsis-associated DIC is described as the systemic activation in coagulation with suppressed fibrinolysis that leads to organ dysfunction in combination with systemic inflammation. Thus, in the context of sepsis, the concept of DIC representing consumptive coagulopathy has been superseded by a more specific approach (“sepsis-induced coagulopathy”; SIC) focusing on the presence of organ dysfunction, decreased platelet count, and increased PT-INR (27).

A large number of different pathogens and their products act on the endothelium via various pathomechanisms. Several predominantly proinflammatory responses of the cell to pathogen-induced stimulation have been identified. In addition to direct pathogen-associated activation, non-specific stimulation of endothelial cells by products of the host response (DAMPs) plays an essential role in the inflammatory process. In the context of some hemorrhagic fevers or acute phases of systemic, exuberant, proinflammatory host response (e.g., sepsis), it is postulated that this damage to the endothelium may be crucial to the course of the disease. In addition, the endothelium contributes significantly to the aggravation of inflammation through the release of proinflammatory substances, recruitment of inflammatory cells, procoagulant activity, and hyperpermeability (28).

Endothelial cells lose their anticoagulant function after proinflammatory stimulation and promote coagulation by decreased expression of thrombomodulin and heparan sulfate on the cell surface and increased expression of tissue factor (TF). Together, increased TF expression by pathogen-activated endothelium, adherent tissue factor-loaded monocytes, and leukocytic microparticles may activate the coagulation cascade. Finally, the pro-inflammatory serine protease thrombin activates the G-protein coupled protease-activated receptor-1 of endothelial cells, enhancing endothelial responses such as hyperpermeability, adhesion molecule expression, and cytokine production (29).

### The Complement System in Sepsis

Complement activation products (such as the anaphylatoxins C3a, C4a, and C5a) are elevated in the early stages of sepsis (30). Physiologically, C5a is associated with the chemotaxis of neutrophils to the site of infection. By binding C5a to the C5a receptor (C5aR), neutrophils develop into migratory cells with the ability to enter inflamed tissue and remove pathogens and debris (31). Here, PAMPs and DAMPs induce the release of NETs, granular enzymes, and reactive oxygen species (ROS) during the oxidative burst, which, in turn, shifts the coagulation balance toward prothrombotic activity, whilst fibrinolysis is inhibited. As a result, disseminated microvascular thrombosis is initiated, and consumption of clotting factors occurs, which is the hallmark of overt DIC (27). An excessive activation of C5a in sepsis causes aggravation of systemic inflammation, progressive apoptosis of lymphocytes, and even dysfunction of neutrophils (32). Overwhelming levels of C5a during sepsis lead to downregulation of C5aR with adverse effects on the further course of the disease. Homing of neutrophils into the microvasculature, further tissue damage, thrombosis, and ultimately multi-organ failure take place. In a mouse model, the blockade of C5a or C5aR inhibits the development of sepsis. Significantly increased survival has been shown in models of mild to moderate sepsis of C5aR-deficient mice, accompanied by improved pathogen clearance and largely preserved liver function (33). In patients with sepsis, however, downregulated levels of C5aR correlate with a poor prognosis when C5a levels are simultaneously elevated (34).

In summary, C5a as well as C5aR are key players in many acute and chronic inflammatory conditions, making C5a a highly attractive pharmacological target. The important involvement in sepsis-related inflammation makes both C5a and C5aR promising starting points for the development of novel therapeutic approaches. With Vilobelimab (anti-complement C5a) and Avdoralimab (anti-receptor C5aR monoclonal antibody; NCT04371367) the respective first-in-class monoclonal antibodies are currently tested both in clinical sepsis trials and in COVID 19 (35).

### Sepsis-Induced Immunosuppression and Persistent Inflammation, Immunosuppression and Catabolism Syndrome

**Figure 2:**
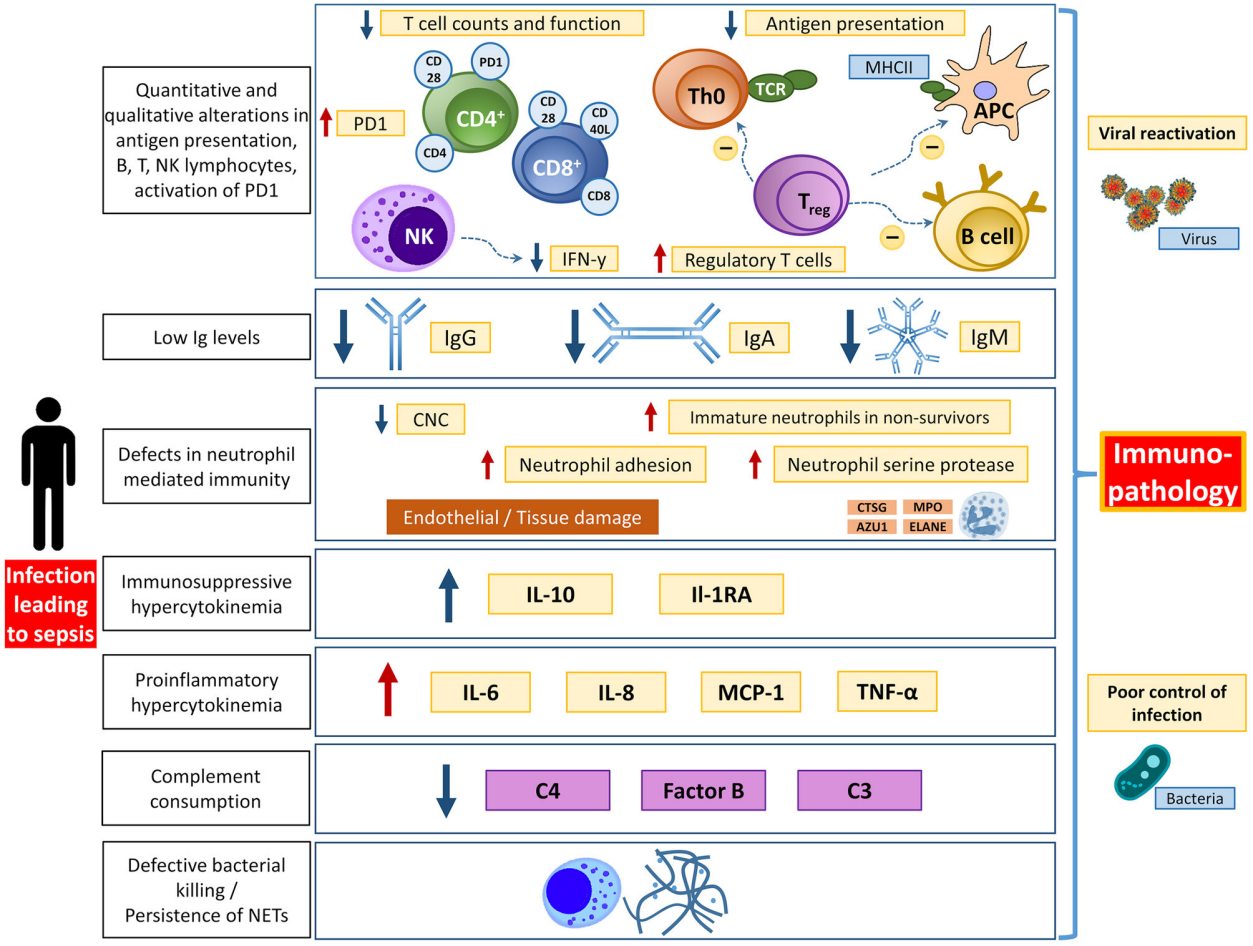
Overview of different aspects of immunological dysfunction with details of the affected entities. APC, antigen presenting cell; AZU1, azurocidine 1; CNC, circulating neutrophils count; CTSG, cathepsin G; ELANE, elastase; IFN-y, interferon y; Ig, immunoglobulin; MHCII, major histocompatibility complex II; MPO, myeloperoxidase; PD1, programmed death protein 1; TCR, T cell receptor. Adapted from Bermejo-Martin JF (12) with permission.

Although the hallmark of sepsis is generally considered to be the early systemic inflammatory response, there is a significant component of immunosuppression that also occurs in both early and late stages of the disease (9, 36–38) (Figure 2). In the early stages of sepsis, the depletion of B and T lymphocytes can be observed in addition to an increased apoptosis rate of stromal cells and APCs (36, 39–44). The mechanisms underlying sepsis-induced lymphopenia are not yet fully understood but may be caused by increased migration into the tissue, increased apoptosis, and reduced production since in emergency hematopoiesis the release of neutrophils and monocytes is given priority (45, 46). The persistence of lymphopenia, and the decrease of immunoglobulin levels during sepsis, are associated with increased mortality (47, 48).

Even though many details about the function of B lymphocytes in sepsis have been revealed, their role goes beyond the production and secretion of immunoglobulins. B lymphocytes also produce cytokines, act as APCs, and modulate the innate immune response (49, 50). Through interaction with dendritic cells, macrophages, T and other B lymphocytes, clonal expansion is induced, which ultimately leads to the synthesis of highly specific antibodies. After differentiation into high-affinity, antibody-secreting plasma cells, B lymphocytes contribute significantly to effective host protection by producing antibodies (51). At the onset of sepsis, B cells can be activated by pathogens directly via interaction with pathogen recognition receptors (PRRs), which leads to an initial immune response by innate-like B cells (49, 52, 53). In septic shock, non-survivors have recently been shown to have pronounced functional impairment of B lymphocytes, resulting in decreased IgM production following stimulation and an overall decreased level of IgM (54). The ratios of different peripheral B cell subgroups (immature/transitional B cells, naive B cells, tissue-like memory B cells, resting memory B cells, and activated memory B cells) in septic shock differ significantly from those of healthy control patients (55). Sepsis survivors also have a significantly higher number of circulating B lymphocytes than non-survivors, especially in the first 24 h after the onset of sepsis (54). This effect can be attributed to the release of IgM, a natural antibody that is particularly important in the fight against Gram-negative bacteria (49). The hypothesis of B lymphocyte protection by secreted IgM is supported by the observation that in survivors of sepsis or septic shock elevated levels of circulating IgM antibodies have been detected in comparison to non-survivors just in the first 24 hours of the disease (54). Interestingly, critically ill patients who did not suffer from septic infection showed a similar picture (56). However, up to now, there is insufficient data justifying the routine use of IgM levels or B lymphocyte counts in the early stages of sepsis for prognostic purposes.

Apart from sepsis-induced lymphopenia, an increased rate of apoptosis of APCs and monocytes is a common observation during sepsis, which is also associated with a significant reduction of pro-inflammatory cytokines (36, 37, 39–44, 57–59). At the same time, there is decreased expression of human leukocyte antigen DR (HLA-DR) on the surface of the remaining monocytes and dendritic cells, resulting in pathogen recognition impairment and a reduction of opsonization with T cell receptor proteins. This leads to disruption of the Th1- and Th2-response as an essential component of the adaptive immune response (60). The inability of monocytes to restore normal levels of HLA-DR expression during the course of the disease has been shown to be a negative predictor for the outcome of sepsis, as well as endotoxin tolerance in the early stages of sepsis (61–63).

In addition to the loss of pro-inflammatory cytokine production due to the reduction of APCs and monocytes, acute infection leads to significantly increased granulopoiesis, whereby immature myeloid cells migrate into the peripheral blood and become functionally active. These myeloid-derived suppressor cells (MDSCs) release anti-inflammatory cytokines (e.g., IL-10 and transforming growth factor ß, TGF-ß), which significantly aggravates immunosuppression (9, 64). In the context of malignant diseases, the immunosuppressive properties of MDSCs are the focus of extensive research. Recently, Darden et al. published the results of a pilot study in which they used single-cell RNAseq to demonstrate different subsets of MDSCs, which are associated with the various courses of sepsis and may thereby be used as prognostic factors (65). Since knowledge of the mechanisms involved in sepsis remains limited, MDSCs seem to be another promising target for future research (66, 67).

In sepsis, the expression of inhibitory immune checkpoint molecules such as programmed death protein 1 (PD-1) is increased on the surface of T cells, APCs and peripheral tissue epithelial cells, which binds to the inhibitory programmed death protein 1-receptor (PD1-R) expressed on B and T lymphocytes (68). Binding to PD1-R suppresses leukocyte function and leads to apoptosis of immune cells, which contributes to the further depletion of T and B cells, APC dysfunction, and expansion of regulatory T cells (T_reg_) (9, 41, 69–72). Although controlled apoptosis of cells of innate and adaptive immunity is initially advantageous for the host, the simultaneous downregulation of the inflammatory response in sepsis leads to the extensive loss of immune cells and the inability of the host to continue to defend itself against invading pathogens. Inhibiting apoptosis of immune cells has been shown to be beneficial in sepsis (73).

In general, acquired immunosuppression in sepsis is caused by epigenetic and metabolic mechanisms resulting in reprogramming of immune cells. After activation of pro-inflammatory genes in early sepsis, histone-mediated alterations lead to conversion of euchromatin to silent heterochromatin (74, 75). These epigenetic processes are linked to metabolic pathways, such as glycolysis or oxidative phosphorylation, which can lead to the accumulation of metabolic products such as acetyl-coenzyme A (Acetyl-CoA) and nicotinamide adenine dinucleotide (NAD) during the course of sepsis. Acetyl-CoA as well as NAD act as cofactors for the epigenetic enzymes histone acetyltransferase and histone deacetylases sirtuin-1which negatively influence gene transcription (73, 76).

In addition, post-translational gene control is provided by non-coding RNA such as microRNA (miRNA). This evolutionarily conserved, non-coding single-stranded RNA plays an important role in gene silencing and in fine tuning of protein expression (77).

miRNAs are usually transcribed and processed within the nucleus through the complex interaction of multiple factors, including RNA polymerase 2, RNase III and the DiGeorge syndrome critical region 8 (DGCR8) complex (78). After shifting into the cytoplasma and final processing steps, miRNA can be guided to messenger RNA (mRNA) for post-transcriptional regulation by the RNA-induced silencing complex (RISC). RISC is a functional conglomerate of RNAse III, transactivation-responsive RNA-binding protein and Argonaute-2 proteins.

Furthermore, also direct interaction to target mRNA via complementary base sequences can lead to functional modification and even degradation of mRNA, thereby adjusting protein expression levels or inhibiting translation. Each mRNA is under the control of numerous miRNAs, and, conversely, each miRNA controls hundreds of mRNAs (79).

miRNA have been detected in several body fluids, such as saliva and urine, but also in plasma. Due to their resistance to temperature, pH and RNAses, miRNA have a system-wide influence on cellular functions (80, 81). The signaling cascade downstream of the activated TLR, e.g., is modified by the action of miRNA so that excessive inflammation in response to an infection is attenuated (82).

Back in 2012, Gentile et al. described the clinical phenotype of persistent inflammation, immunosuppression, and catabolism syndrome (PICS) in surgical patients with a prolonged (>10 days) ICU stay (83). This condition, which had previously been described as “late MOF,” “CARS,” or “complicated clinical course,” typically evolves after an initial and pronounced septic or inflammatory insult. It is characterized by persistent inflammation and acquired immunosuppression, prolonged ICU stay, and is usually associated with poor outcome. PICS may also occur after major trauma, and in elderly patients with sarcopenia and immunosenescence experiencing trauma, major surgery, or sepsis (84). Early diagnosis and advanced organ support in sepsis have substantially decreased mortality for many patients admitted to the ICU. However, a significant proportion of sepsis survivors develops chronic critical illness (CCI) with ongoing organ dysfunction. A subset of CCI patients will develop PICS, predisposed to a poor quality of life and indolent death (85). With an aging population in many developed countries, many sepsis survivors will eventually develop CCI after successful initial resuscitation, which is a debilitating condition with profound personal and social costs.

### Acute Respiratory Distress Syndrome in Sepsis

When critically ill patients develop pulmonary dysfunction, there is often an associated primary pulmonary affection, such as pneumonia, exacerbation of chronic obstructive pulmonary disease (COPD), aspiration, pulmonary embolism, or pulmonary contusion. A progression of lung injury often leads to acute respiratory distress syndrome (ARDS), as defined by the Berlin criteria (86). In septic shock, the incidence of severe ARDS is reported to be up to 40%, and is more frequent in the presence of a pulmonary focus (87, 88). The occurrence of ARDS may also lead to the development of dysfunction in other organs, such as kidneys, liver, cardiovascular and central nervous system, which often persists until late in the course of the disease.

In case of sepsis induced multiple organ dysfunction syndrome (MODS), the lungs are the predominant organ system affected, and a primary pulmonary pathology is absent in many cases. Sepsis-induced ARDS is caused by an uncontrolled and complex interaction between inflammatory cytokines and cellular mediators that damage the alveocapillary unit and can be classified in three overlapping phases (89):

- exudative phase, characterized by edema and alveolar hemorrhage within the first days.
- proliferative phase, marked by organization and repair.
- fibrotic phase, usually after 3–4 weeks after the onset of ARDS and characterized by collagenous fibrosis.

Direct or indirect damage to the pulmonary epi- and endothelium leads to increased alveolar capillary permeability, resulting in progressive exudate of protein-rich fluid. Plasma proteins in this fluid inactivate surfactant factor, and *de-novo* production of surfactant is reduced by ongoing damage to type 2 pneumocytes. The resulting surfactant deficiency leads to an increase in intraalveolar surface tension, thus causing diffuse microatelectasis (90). Additional injury to the alveolar capillary membranes is exacerbated by neutrophil entrapment in the pulmonary microcirculation. The local release of inflammatory mediators by neutrophils and macrophages migrating into the alveoli and interstitial space contributes to diffuse endothelial cell injury and destruction. In parallel, there is deposition of leukocytes and platelets and progressive destruction of type I alveolar pneumocytes, whereas type II alveolar pneumocytes show hyperplasia. In the advanced stage of these changes, a morphological condition called diffuse alveolar damage (DAD) develops (91).

If successfully addressed at an early stage, lung injury is almost fully reversible. However, if there is a persistent exudate of protein-rich fluid and further infiltration by neutrophils, mononuclear cells, fibroblasts and lymphocytes, respiratory failure progresses, and pulmonary fibrosis completely transforms the lungs. Collagen is accumulated and microcystic honeycomb, traction bronchiectasis and fibrosis of the alveolar ducts occur as well as abnormally enlarged air spaces with an abnormal increase in dead space (92).

### Sepsis-Induced Acute Kidney Injury

The pathophysiology of the development of sepsis-associated acute kidney injury (sa-AKI) is still poorly understood. Progress in research is slow and often based on extrapolations from postmortem observations, cell cultures, and animal models. The prevailing pathophysiological concept primarily identifies decreased renal blood flow resulting in tubular epithelial cell necrosis as the probable cause for AKI as a consequence of hypoperfusion and shock (93, 94). However, recent findings suggest that not only hypoperfusion but also other factors must play a role: sa-AKI may both occur in stable (macro)circulatory conditions and during increased renal blood flow (95). Histopathological findings of postmortem human and animal tissue samples do not allow to draw a direct line between severity of renal parenchymal damage and functional changes. These observations lead to the conclusion that different mechanisms are involved in the development of sa-AKI.

Sepsis is usually accompanied by the co-occurrence of inflammation, microcirculatory dysfunction, and metabolic reprogramming. Inflammatory mediators, DAMPs and PAMPs are released into the intravascular space and, upon binding to membrane-bound receptors such as TLR, cause the synthesis and release of additional proinflammatory molecules. Specific subtypes of these receptors, such as TLR-2 and TLR-4, are also expressed by renal tubular epithelial cells (TECs). Here, binding of damage- or pathogen-associated molecular patterns cause an increase in oxidative stress and mitochondrial damage. Paracrine signaling also occurs in an attempt to protect neighboring cells from damage by inactivation, but this also leads to a decline in organ function. In addition to endotheliopathy and glycocalyx damage, activation of the coagulation cascade and autonomic nervous system signaling can also impair the microcirculation (96, 97). This results in functional occlusion of capillaries by leukocytes and platelets, and damage to the endothelium is accompanied by vasodilation and endothelial leakage. The resulting edematous peritubular distension reduces the oxidative supply to TEC due to the prolonged diffusion distance. The mechanisms described above in the course of sepsis lead to an intrarenal redistribution of blood flow, with hypoperfusion of the renal medulla.

Early diagnosis and initiation of appropriate therapeutic measures in sa-AKI is time critical. Advanced and more sensitive markers for kidney damage or AKI risc prediction are needed, such as neutrophil gelatinase-associated lipocalin (NGAL), urinary kidney injury molecule-1 (KIM-1) and the combination of the regulatory proteins IGFBP7 and TIMP-2 (98, 99). NGAL is released by renal TECs and was shown in early studies to be a good tool for predicting RRT need and in-hospital mortality. However, because NGAL is also released by activated neutrophils, it may also be elevated in non-renal causes (100, 101). KIM-1 is released from proximal TECs after nephrotoxic and ischemic damage and was also shown to be a good predictor of AKI. In sepsis, survivors versus non-survivors had significantly lower KIM-1 levels at 24 and 48 h. In a study of 86 children with circulatory collapse, KIM-1 was able to predict impending AKI before changes in serum creatinine as well as GFR (102, 103). So far, these promising approaches with NGAL and KIM-1 have not been proven in sufficiently powered clinical trials. The combination of urinary IGFBP7 and TIMP-2 showed good prediction of AKI in several studies, with non-renal organ failures not leading to a change in their levels (104–106). As regulatory proteins of G1 cell cycle arrest, both have a protective effect during cellular stress and quantify an individual patient's risk for impending AKI (105). Because of its involvement in endothelial dysfunction and capillary leakage, mid-regional proadrenomedullin (MR-proADM) is also of great interest. In a secondary analysis of SISPCT trial, which enrolled more than thousand severe sepsis and septic shock patients, it was shown that MR-proADM within the first seven days of sepsis provided a more accurate prediction for requirement for RRT than urine output and creatinine (107, 108). These results could recently be confirmed in patients with COVID-19 by our group, suggesting that MR-proADM may be a useful predictor for requirement of RRT during ICU stay (109).

### Cardiac Dysfunction

The term sepsis-induced cardiac dysfunction is used to describe a variety of acute cardiac disorders caused by sepsis. Septic cardiomyopathy has become the focus of much research in recent decades and is associated with significantly increased mortality of up to 50% (110). Septic cardiomyopathy represents a complex cardiac dysfunction affecting both ventricles. Apart from non-specific conditions (age, obesity), no specific risk factors are known to date. Clinically, it presents with all the signs of circulatory failure associated with systemic infection. The differences to the clinical manifestation of cardiac dysfunction in patients with decompensated heart failure of non-septic etiology lie in the features of altered global hemodynamic parameters (preload, afterload, microcirculation). Unlike other myocardial pathologies, septic cardiomyopathy therefore requires a multimodal approach to diagnosis and therapy.

The underlying pathophysiological mechanisms can be broadly classified into three groups: impaired myocardial circulation, direct cardiac depression, and impaired cardiac mitochondrial function (111).

A balanced intravascular fluid status is a major prerequisite for cardiac function, and loss of vascular tone due to arterial vasodilation is one of the main causes of hemodynamic instability secondary to sepsis. The development of sepsis-induced endothelial dysfunction also plays a major role. A mere maintenance of coronary blood flow does not protect against the development of myocardial dysfunction, since endothelial damage may cause profound microcirculatory maldistribution of blood flow (112).

Direct myocardial depression is based partly on sepsis-related decrease in myocardial adrenergic response due to downregulation of ß-adrenergic receptors and their components, caused by pro-inflammatory mediators. In particular, IL-1ß and TNF-α appear to have a pronounced direct effect on myocardial contractility *in vitro* (113). Il-1 stimulates increased synthesis of nitric oxide (NO) via NO synthase (NOS) and thereby enhances its effects in the cardiovascular system (114, 115).

Due to the effect of NO on both cardiac ß-adrenergic receptors (where it leads to suppression of adrenergic response) and on mitochondria (inducing functional impairment), NO was shown to be associated with the severity of cardiac dysfunction and with increased mortality (116). Further, increased levels of prostanoids (e.g., prostacyclin and thromboxane) appear to influence coronary endothelial function. Therapeutic approaches to reduce the effect of prostanoids by using anti-inflammatory agents such as cyclooxygenase inhibitors (e.g., ibuprofen, indomethacin) have not shown efficacy in clinical studies.

Another mechanism of sepsis-induced cardiac dysfunction is the influence of the complement system. Activation of complement factor C5 (C5a) as a strong proinflammatory mediator promotes the release of granular enzymes, the release of further cytokines and ROS and increases chemotaxis of neutrophils. In addition, cardiomyocyte-expressed C5a-receptors mediate further C5a-induced cardiodepression, thereby making it a potential target for anti-C5a antibodies (117).

Since the beating heart is highly dependent on the continuous supply of ATP, metabolic dysfunction of myocardial mitochondria has also been identified as a key mechanism in the development of septic cardiomyopathy (118, 119). Cardiomyocytes contain a large number of mitochondria, which explains the close relationship between sepsis-induced cardiac dysfunction and outcome in case of mitochondrial dysfunction (120). Increasing levels of ROS and reactive nitrogen species (RNS) have a negative impact on oxidative phosphorylation and directly inhibit mitochondrial respiration, which may lead to apoptosis in addition to other direct damage to cellular components (121, 122). Consumption and deficiency of antioxidants (ascorbic acid, a-tocopherol, uric acid, and others) during sepsis may amplify oxidative stress and are associated with the development of organ failure in sepsis. However, none of these agents so far have been convincingly demonstrated to bring about clinically meaningful benefits (123–125).

A balanced distribution of Ca^2+^ is fundamentally linked to the regeneration of ATP. However, cytokines and other mediators lead to Ca^2+^ overload of mitochondria in sepsis by disrupting the Ca^2+^ storage function of the sarcoplasmic reticulum. Ca^2+^ overload leads to the opening of mitochondrial permeability transition pores (mPTPs) and subsequently to caspase protein-induced mitochondrial damage (126, 127). With the assistance of mPTPs, circular mitochondrial DNA (mtDNA) can be released from mitochondria (128). Acting as a DAMP, mtDNA can activate the immune response via TLR-9, and plasma levels have been shown to be significantly lower in sepsis survivors than in non-survivors (129–131).

Although sepsis-induced cardiac dysfunction is closely related to prognosis and has been the focus of many research projects, effective treatment options are lacking.

### The Role of Immunoglobulins

Immunoglobulins are produced and released by differentiated B cells (plasma cells) (132). The variable regions of these glycoproteins allow non-covalent cross-linking with bacterial and other antigens, whereby the constant region signals antigen binding. Within the human humoral immune system, IgA, IgG, and IgM are the most important classes. The main function of IgA is mucosal immunity, while IgG brings about opsonisation and complement activation in addition to secondary antibody reactions. In addition to the primary antibody response, the main function of IgM is complement activation. The antigen binding affinity of natural IgM antibodies is typically lower when compared to IgG, but their polyvalence allows for high avidity binding and efficient engagement of complement to induce complement dependent cell lysis (133). IgA, IgG, and IgM are known to behave synergistically in sepsis and septic shock, and the simultaneous occurrence of low plasma levels of these antibodies is associated with reduced survival in patients at the onset or during sepsis or septic shock (54, 134–141). The etiology of low plasma levels of immunoglobulins in sepsis is not fully understood but is most likely the result of multifactorial events such as endothelial dysfunction with subsequent vascular leakage, redistribution to inflamed tissue, complement consumption, excessive catabolism, and downregulated production and secretion due to secondary immunosuppression (12, 132, 135, 136, 142).

## Therapeutic Concepts in Sepsis and Septic Shock

In 2004, the Surviving Sepsis Campaign (SSC) was initiated as a campaign to advance the worldwide treatment of sepsis comprehensively to improve survival through joint efforts. From the beginning, “sepsis bundles,” i.e., a set of procedural measures to be taken within a prescribed time window, have been the cornerstones for the successful treatment of sepsis and septic shock. Strict adherence to and consistent application of these bundles have reduced the relative risk of mortality by up to 25%, although the evidence for the efficacy of individual measures remains controversial (4, 143, 144).

Today, standard therapy for sepsis consists mainly of attempting to eliminate the focus like interventional radiology or surgical measures for source control and timely administration of empirically targeted antibiotics (causal therapy). Further, additional intensive care measures are used for individual organ support like vasopressor administration, mechanical ventilation, and renal replacement therapy (supportive therapy). Parallel to these standards, adjunctive treatments can be used.

### Causal Therapy

It is widely accepted that an early start of interventions is crucial for success. The “Hour-1-Bundle” was introduced in response to new evidence based on the 2016 guidelines and replaces the previous recommendations of the 3- and 6- h bundles with the explicit intention of starting fluid resuscitation and sepsis management measures immediately. The “Hour-1-Bundle” consists of 5 clinical interventions: blood cultures prior to antibiotics, administration of broad-spectrum antibiotics, administration of IV fluid, application of vasopressors, and measurement of lactate levels.

Aiming to eliminate the source of infection as the underlying cause of the continuing immune imbalance is of fundamental importance for successful sepsis therapy. If this fails or is incomplete, the probability of survival will be reduced (145). In addition to surgical interventions, the removal of existing intravascular catheters and devices should also be considered. Blood cultures and other biologic samples should be obtained, but this should not delay antimicrobial therapy (146). The causal treatment of a (suspected) underlying infection and hemodynamic management must be performed simultaneously. In 2014, the MEDUSA trial showed that delay in antimicrobial therapy and source control was associated with increased mortality in sepsis and septic shock patients (147).

The second mainstay in the therapy of sepsis and septic shock is antibiotic therapy. For this, the best possible knowledge of the pathogen epidemiology and the presumed anatomical focus are prognostically important. Due to the substantial variability of pathogens between different countries, regions and hospitals, the local pathogen and resistance situation should be known, and regular updates should be scheduled.

Broad-spectrum antibiotics, which empirically cover the expected pathogen spectrum, are the first choice and should be administered at the earliest possible point in time. However, to what extent does the classification of the pharmaceutical agents matter, does it make a difference whether bactericidal or bacteriostatic drugs are chosen? And what are the differences between these two categories? Contrary to what is implied by the literal designation, it is rather a matter of definition. To classify an antibiotic agent, knowledge of two parameters is essential. First, the minimum inhibitory concentration (MIC), which defines the concentration of agent that prevents the visible growth of bacteria under defined conditions (e.g., growth media, temperature, CO_2_ concentration). Second, the minimum bactericidal concentration (MBC) of the agent, which that results in a 1,000-fold reduction in bacterial density within 24 h under the same specific conditions. Based on the ratio of MIC and MBC, the classification of the respective antibiotic agent is formally defined: a ratio of MBC-to-MIC >4 is determined bacteriostatic, whilst a ratio MBC-to-MIC ≤4 is determined bactericidal (148). In a systematic review of a total of 56 randomized controlled trials (RCTs) published between 1985 and 2017 comparing bactericidal and bacteriostatic antibiotics, Wald-Dickler et al. found no evidence for the superiority of bactericidal antibiotics. Of the evaluated RCTs, which investigated a broad spectrum of underlying infections, six showed a statistically significant difference in favor of bacteriostatic agents, and only one publication showed an advantage for a bactericidal antibiotic (however, the bacteriostatic antibiotic was dosed suboptimally) (149). No compelling link to the clinical course of infection and no prediction with regard to outcome could be identified, as long as the choice of the agents used and their respective dosage was based on evidence-based data.

Following an initial loading dose, further dosage of antimicrobial agents should be adapted according to drug properties and pharmacokinetics as well as pharmacodynamic principles, since the function of almost every organ system is significantly disturbed during sepsis or septic shock (increased distribution volume due to aggressive fluid resuscitation and capillary leakage, hypotension, restricted renal and hepatic function). As soon as the causative pathogen is identified, narrowing of the initial therapy is recommended (150).

#### Use of Lactate as Marker for Severity and Disease Progression

Lactate production and clearance are influenced by numerous factors. In the context of DO_2_/VO_2_ mismatch during critical illness, elevated lactate levels can have various causes, such as an anaerobic metabolic response due to tissue hypoxia, decreased hepatic clearance, or pronounced ß-adrenergic stimulation of Na/K-ATPase leading to a consecutive increase in aerobic glycolysis (151). A persistent elevation of serum-lactate above 2 mmol/l in critically ill patients is an independent predictor of mortality across different groups of ICU patients with sepsis, trauma, organ failure, and shock due to septic, cardiogenic, and hemorrhagic etiology, among others (152). A retrospective analysis of 400 patients with severe hyperlactatemia (>10 mmol/l) showed markedly increased ICU mortality compared with the overall cohort of ICU patients, with wide variation in mortality among the different groups (153). If severe hyperlactatemia persisted for more than 24 h, it was associated with extremely high mortality (>95%); if it lasted more than 48 h, none of the patients survived. In a recent evaluation of 781 ICU patients, Hayashi et al. showed that maximum arterial lactate concentration within 24 h provided robust prediction of both in-hospital mortality and 90-day survival, comparable to the predictive power of APACHE III in unselected ICU patients (154).

Due to its availability and strong association with disease severity and patient outcome, lactate has an outstanding role as a diagnostic marker and as a marker of disease progression. This holds true for both the absolute values and for the kinetics over time (lactate clearance) (155). It is also part of the current “sepsis-3” definition, where a lactate value >2 mmol/l despite adequate volume substitution in conjunction with the need for vasopressor therapy to maintain a mean arterial pressure ≥65 mmHg defines septic shock. Because of its easy availability, close monitoring of the lactate value whilst >2 mmol/l (e.g., every 1–2 h) is a recommended parameter to guide volume therapy and hemodynamic management (146, 156).

### Supportive Therapy

#### Fluid Resuscitation

An essential component of the “Hour-1-bundle” is adequate volume therapy to treat sepsis-induced tissue hypoperfusion and to counteract absolute and/or relative hypovolemia caused by vasodilatation, external fluid loss and capillary leakage. Immediately after identification of a septic patient with hypotension and/or elevated lactate levels, the treatment should be initiated. This has been repeatedly shown to reduce mortality (157, 158). Following the current paradigm, 20–40 ml/kg of crystalloid fluid should be administered within the first 3 h in accordance with SSC guidelines. The use of fluids other than crystalloids for initial resuscitation and intravascular volume replacement is currently not recommended in patients with sepsis and septic shock. If hypotension persists despite adequate fluid resuscitation, the use of catecholamines is indicated to ensure adequate perfusion of vital organs and to maintain mean arterial pressure above 65 mmHg. The guidelines recommend norepinephrine as the vasopressor of choice, according to current data, with moderate evidence.

Although there is general consensus that high-dose fluid replacement is indicated in the first hours of septic shock for rapid normalization of oxygen delivery and circulatory function, there is general uncertainty about the further continuation of fluid administration and appropriate target parameters. There is increasing concern that a continued positive fluid balance will have a negative impact on prognosis (159–162). At the same time, there is evidence that significantly earlier use of vasopressors may be beneficial, contrary to what is recommended by current guidelines (163, 164). At what point vasopressors should be started and fluid therapy stopped is still unclear. Thus, prospective studies evaluating the impact of early vasopressor therapy on the development of multiorgan dysfunction and the total volume of resuscitation fluids required during early septic shock are clearly needed.

#### Steroids

The use of corticosteroids as a supportive measure in the treatment of sepsis and septic shock has been a matter of debate for decades. Currently, SSC guidelines recommend the use of hydrocortisone only in patients with vasopressor-dependent refractory septic shock, who do not respond to fluid resuscitation. It is recognized that there are no data demonstrating a survival benefit from continued use of hydrocortisone in sepsis therapy. In 2008, a European multi-center study in a cohort of almost 500 patients showed no improvement in 28-days mortality when using hydrocortisone in septic shock. In addition to an increase in secondary infections, an increased incidence of hypernatremia and hyperglycemia was observed, and as a result, hydrocortisone was no longer recommended as standard therapy in septic shock (165). In 2018, the influence of adjuvant glucocorticoid therapy on 90-days mortality was investigated in 3,800 patients with septic shock (ADRENAL). Although there was a more rapid hemodynamic stabilization and also a shortening of the duration of mechanical ventilation, no significant difference in 90-days mortality was found (166). However, the results of yet another study (APROCCHSS) from 2018 partially contradict these findings. Annane et al. were able to demonstrate a significant reduction in 90-days mortality (43.0 vs. 49.1%). *p* = 0.03) in favor of intervention when hydrocortisone plus fludrocortisone was used in adult septic shock patients, but the study population differed from the first cohort with a lower proportion of surgical patients, abdominal infections and a higher proportion of renal replacement procedures (167). In 2017, Marik et al. demonstrated within a retrospective before-after study that moderate doses of hydrocortisone in combination with early administration of IV vitamin C and thiamine can effectively prevent progressive organ dysfunction, including acute kidney injury. Compared to a control cohort, they demonstrated a dramatic reduction in mortality in patients with sepsis and septic shock (8.5 vs. 40.4%, *p* < 0.001). Unfortunately, these promising results could neither be confirmed in a further retrospective evaluation nor in a multicenter randomized open-label study after enrollment of 216 patients with septic shock. No significant change in survival or vasopressor-free time over 7 days could be demonstrated after a triple therapy of vitamin C, thiamine and hydrocortisone vs. hydrocortisone alone (168, 169). In toxic shock syndrome (TSS) caused by staphylococcal or streptococcal exotoxins acting as superantigens, the use of steroids is not recommended; there is only anecdotal and outdated evidence for beneficial effects (170, 171). However, the administration of IVIG in TSS has repeatedly been suggested (cf. 3.3.4 Immunoglobulins in sepsis).

#### Ventilation

In the field of mechanical ventilation as a supportive measure for patients with sepsis and respiratory insufficiency, only moderate progress has been achieved so far. The goals of mechanical ventilation include improving gas exchange and reducing work of breathing, as well as preventing high airway pressures and further iatrogenic damage to the lung tissue. In sepsis-induced acute respiratory distress syndrome (ARDS), the recommendation for lung-protective ventilation with a tidal volume of 6 ml/kg standard body weight with an upper limit for the plateau pressure of 30 cm H_2_O remains valid. Prone position in ARDS is also highly recommended and recognized to lower mortality. Despite low complication rates, a significant reduction in driving pressure and an improvement in oxygenation, an international observational prevalence study showed in 2018 that prone positioning was only used in 32.9% of patients with severe ARDS (172, 173).

Meduri et al. demonstrated in an RCT in patients with refractory ARDS that prolonged administration of methylprednisolone was associated with clinical improvement and reduced mortality (174). The rate of infections was comparable in the study groups. Overall, the evidence for the regular use of steroids in ARDS is still insufficient.

### Adjunctive Therapies

Over the last decades, the knowledge about the manifold and complex immunological interactions, the pro- and anti-inflammatory pathways as well as the disorders of the complement and coagulation system has improved. Unfortunately, however, it has not been possible to translate this knowledge into therapeutic approaches for the treatment of sepsis. Many of the common procedures used in daily intensive care medicine cannot be considered to be fully based on criteria of evidence-based medicine so that up to date, no adjuvant therapy for sepsis and septic shock has proven to be effective for sepsis and septic shock (9, 175).

#### Extracorporeal Blood Purification

**Figure 3:**
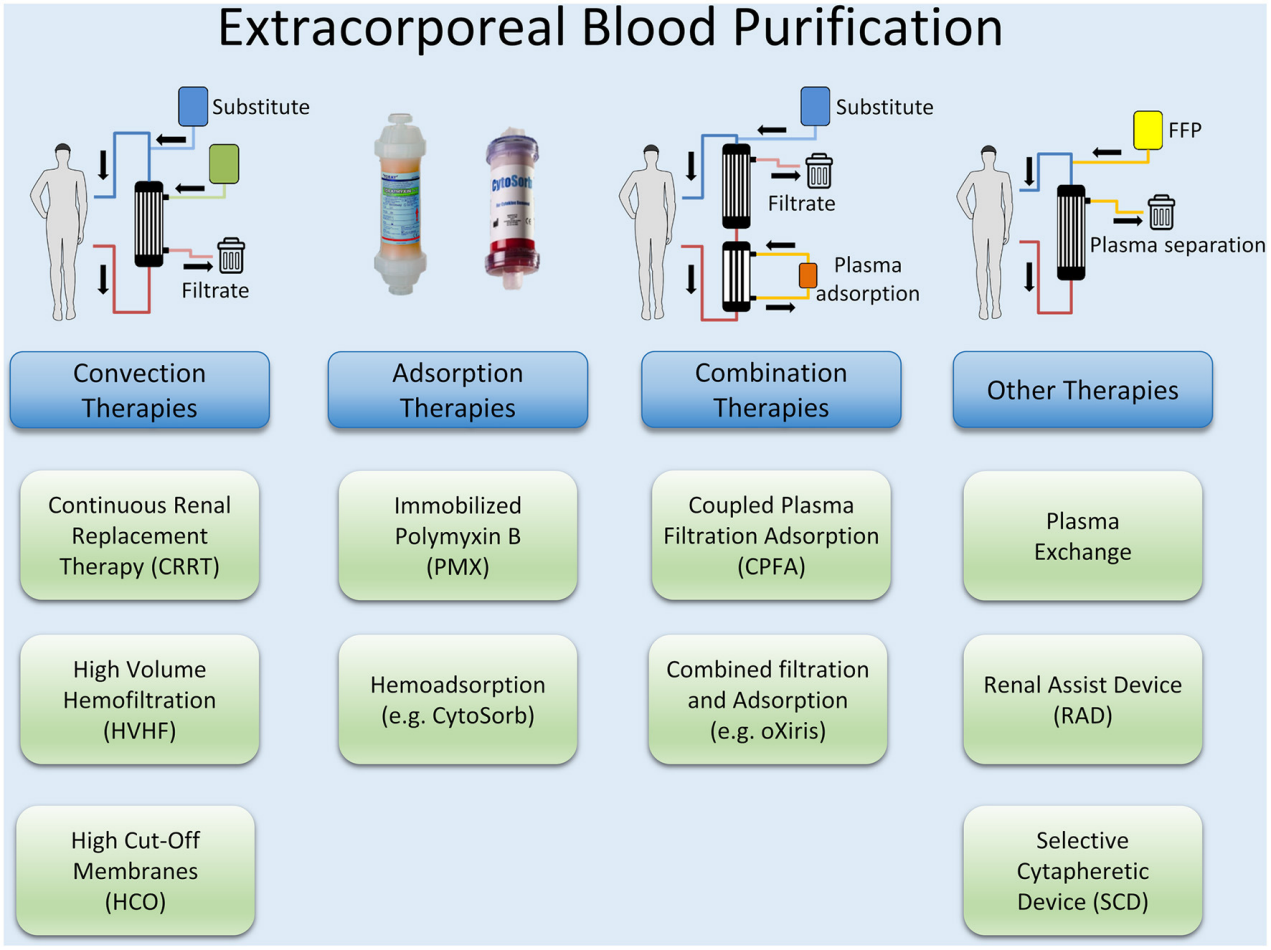
Currently available blood purification methods.

Besides preventing a continued activation of the pro- and anti-inflammatory pathways by an early reduction of PAMPs and DAMPs with source control measures, controlling excessive levels of cytokines and mediators by blood purification methods may be a reasonable approach. Extracorporeal blood purification techniques (BPTs) consist of different approaches and methods, most of which have their origin in renal replacement therapy (RRT). Examples are high volume hemofiltration (HVHF) and plasmapheresis, but also the use of special filters such as high cut-off (HCO) membranes and methods for the adsorption of endotoxin and cytokines or combinations of these methods such as coupled plasma filtration adsorption (CPFA). An overview of different techniques of extracorporeal blood purification is illustrated in Figure 3.

Although extracorporeal blood purification therapies have been shown to remove both inflammatory mediators and bacterial toxins, there is still a lack of evidence for their efficacy in sepsis therapy (176). Technically, HVHF does not differ from conventional RRT, as no additional components have to be added to the circuit. However, an increased convective target dose of well above 35 ml/kg/h is used. The procedure is easy to use if experience in the implementation of continuous renal replacement therapies is available. With HVHF, inflammatory mediators are removed from the bloodstream by convection. The effect on the outcome of sepsis and septic shock has been investigated in numerous studies, in which different convective target doses and continuous vs. ntermittent application were examined (177–180). A recent meta-analysis could demonstrate both hemodynamic improvement (lower HR and higher MAP), and lower mortality of critically ill patients, but no substantial influence on oxygenation Index or disease severity. Also, most RCTs included in the meta-analysis were not of high quality and there was no uniform observation period concerning mortality (181).

The use of high-cut-off (HCO) membranes with an increased pore size (20 nm vs. 10 nm for the standard high-flux membrane) should offer a more effective elimination of inflammatory mediators. In one clinical trial, improved elimination of the inflammatory mediators IL-1, IL-6, and TNF-α was demonstrated in patients with sepsis-induced renal failure, but at the same time there was also significant albumin loss (182). Other studies were terminated prematurely due to the lack of difference in 28-days mortality, vasopressor requirements, ventilation days and ICU length of stay compared to conventional membranes (176).

Recently, there has been renewed interest in plasmapheresis for patients with severe refractory septic shock, the suggested rationale being (apart from blood purification) a rapid substitution of consumed protective plasmatic factors to support microvascular barrier function and microcirculation (177). To date, however, only sparse data are available on the use of therapeutic plasma exchange (TPE) in sepsis. In a recent meta-analysis, Putzu et al. showed that the use of plasmapheresis was associated with decreased mortality compared with standard therapy (183). In addition to a recent pilot RCT with 40 patients, which demonstrated a reduction in catecholamine requirements in patients with septic shock, the EXCHANGE trial is another prospective multicenter study with 352 participants, which investigates the efficacy of therapeutic plasma exchange in septic shock (NCT03065751) (184).

Various proteins and receptors balance the interaction between the endothelium of the vessels and circulating cells. Von Willebrand factor (VWF), with its multimeric structure, is a key protein in platelet-vessel wall interaction. The sensitive balance is controlled by a disintegrin and metalloproteinase with a thrombospondin type 1 motif, member 13 (ADAMTS-13, also known as von Willebrand factor-cleaving protease; VWFCP). Reduced ADAMTS-13 activity can lead to markedly elevated levels of large VWF multimers, resulting in thrombocytic microangiopathy (TMA). Extreme but also typical forms of this are thrombocytopenic thrombotic purpura (TTP) and thrombocytopenia-associated multiple-organ failure (TAMOF). Sepsis is often associated with ADAMTS-13 deficiency due to immune-mediated antibodies, and the severity of this deficiency appears to be associated with outcome (185, 186). In addition to replacing ADAMTS-13 with recombinant proteins, the therapeutic armamentarium also consists of TPE, potentially eliminating circulating pathogens or (auto-) antibodies in addition to replacing missing or depleted proteins (187, 188).

Coupled plasma filtration adsorption (CPFA), which was developed as a treatment for sepsis in the 1990s, is a combination of blood purification methods (189). After separation of plasma from cellular blood components with a highly permeable filter, adsorption within the plasma component by styrene polymer resin is performed before the purified plasma is returned to the cellular components and subjected to conventional hemofiltration. By avoiding direct contact between blood cells and the adsorption material, improved biocompatibility is described (190). The largest RCT to date, with 192 patients, was terminated prematurely in 2014 due to futility, without demonstrating any difference in terms of hospital mortality or ICU-free days (191). The follow-up studies COMPACT 2 (NCT01639664) and ROMPA (NCT02357433) were terminated prematurely in 2017, since the COMPACT 2 study detected a significantly increased mortality for the therapy group within the first 72 h after enrolment (192). This finding ultimately led to the discontinuation of ROMPA. At this time, no further studies are known to investigate the effect of plasmapheresis in sepsis therapy.

#### Adsorption Techniques

In Gram-negative sepsis, endotoxin (lipopolysaccharide (LPS) and its fragments trigger the activation of different cell types (monocytes, endothelial cells, polymorphonuclear neutrophils, and tissue-resident cells) and plasma systems (complement and coagulation pathways). It seemed logical to devise extracorporeal systems that could remove the triggering stimulus. Polymyxin B, a cyclic lipophilic peptide antibiotic, is the ligand most studied for neutralizing LPS because of its high affinity for the lipid A moiety in endotoxin. Two randomized controlled trials have evaluated a device using hemoperfusion through polymyxin B-immobilized fiber columns (PMX) in sepsis or septic shock with abdominal focus, but have shown contradictory results in terms of mortality reduction: Cruz et al. (2009) showed a trend toward mortality reduction, however, this result could not be confirmed by Payen et al. (193, 194). In another clinical trial the impact on mortality in patients in septic shock and high endotoxemia should also be investigated (195). However, after enrolment of 450 patients and completion of the study, it was shown that the primary endpoint of 28-days mortality was not reached by “per-protocol analysis” (196). A subsequent *post-hoc* analysis of the data revealed that patients with high endotoxin levels had a significant reduction in mortality, significant improvements in mean arterial pressure and an increase in ventilator-free days (197). Further evaluation of the data suggests that there may be an upper limit of endotoxin load for successful treatment with PMX.

Besides specific adsorption of endotoxin in Gram-negative sepsis, a broader approach of cytokine adsorption might be more promising. Hemadsorption using the CytoSorb® adsorber column is a non-selective and concentration-dependent method by which a spectrum of cytokines and inflammatory mediators like IL-1β, IL-6, IL-8, IL-10, and TNF-α are adsorbed from the bloodstream. In addition, free hemoglobin, myoglobin, bilirubin, bile acids and bacterial toxins (except endotoxin), activated complement and some drugs are eliminated. This technique could therefore be a suitable approach in the context of an excessive pro-inflammatory and anti-inflammatory response, especially in the early phase of sepsis (198–200). Despite widespread clinical use, the available evidence for this technique showing a positive impact on outcome in septic patients is still limited.

In 2017, a prospective single-center study with 20 consecutive patients with refractory septic shock was published in which cytokine adsorption was used as a rescue therapy (201). The study showed a significant reduction of vasopressor requirement and an increase of lactate clearance resulting in the resolution of septic shock in 13 patients (65%). Another case series of 26 patients with septic shock and renal replacement therapy also demonstrated that cytokine adsorption was associated with rapid stabilization of hemodynamic parameters, a reduced need for vasopressors, and a reduction of serum lactate (202). Compared to mortality prediction by the APACHE II score (Acute Physiology And Chronic Health Evaluation II), this study showed reduced observed mortality for patients in whom cytokine adsorption was initiated within 24 h after onset of sepsis, however, no control group was included.

In 2015 an international registry was established to evaluate the use of cytokine adsorption under real-life conditions. According to the last published interim evaluation after enrolment of more than 600 critically ill patients, in 60% of these patients the indication to use CytoSorb^®^ was sepsis and septic shock (203). Analysis of the completed patient data sets (*n* = 495) showed a significant reduction in IL-6 levels and an observed 28-days mortality of 62.5% compared to an expected mortality rate of 71.3% as predicted by APACHE-II. There was a trend indicating that patients with the highest disease severity benefitted most from the intervention. Further, no adverse events were recorded in septic patients. The inherent absence of a control cohort and patient heterogeneity are reasons why these results cannot uncritically be adopted into clinical practice. Further randomized controlled trials are currently underway in patients with sepsis and septic shock, but also in patients with severe COVID-19.

#### Immunotherapy

One of the main causes of the high mortality in intensive care units continues to be sepsis-induced immunosuppression. As a hallmark, there is often a remarkable reduction in the number of circulating lymphocytes, including CD4^+^- and CD8^+^- T cells and B cells at the onset of sepsis, which lasts up to 28 days and is significantly correlated with mortality (40, 47). Major advances in proteomics, metabolomics and genomics as well as in point-of-care diagnostics enable a novel approach in the therapy of sepsis linked to the term “-OMICS” (204). Successful interventions to influence and reposition the immune system of the host with the help of immunomodulating substances might be transferred from cancer therapy, where the application of immunomodulating therapies is already part of the therapeutic armamentarium (205).

Antibodies against programmed cell death 1 (PD-1) receptor and the corresponding ligand (PD-L1) as well as Interleukin-7 are considered promising candidates for the treatment of sepsis and its immunological consequences. PD-1 and PD-L1 modulate as key components (“checkpoints”) in a negative costimulatory pathway the duration and amplitude of the normal T cell immune response toward infectious stimuli. The activation of PD-1 enhances immunosuppressive signals and reduces effector function in both the innate and adaptive immune system (206). While PD-1 is only expressed on activated immune cells such as T cells, PD-L1 is expressed by a variety of other cells like antigen-presenting cells and tumor cells. The binding of PD-1 to PD-L1 results in a reduced release of cytokines, a growth arrest of T cells, and even to apoptosis. Exhausted T cells regularly show surface expression of PD-1 and PD-L1. Increased expression of PD-1 and PD-L1 is also found on circulating monocytes and CD4^+^ lymphocytes in patients in septic shock, which is associated with the occurrence of secondary (nosocomial) infections and increased mortality (69, 207).

In patients with sepsis-related immunosuppression, addressing PD-1 and/or PD-L1 appears to be an option with potential clinical benefit, especially since checkpoint inhibitors have already been successfully used in cancer immunotherapy (208). In *ex-vivo* studies in human cells, the use of monoclonal antibodies blocking either PD-1 or PD-L1 led to an increase of cytokine production and secretion by T cells and monocytes (209). Nivolumab is a human IgG4 monoclonal antibody that binds to the programmed cell death 1 (PD-1) receptor and prevents the receptor from interacting with its PD-L1 and PD-L2 ligands. Nivolumab has been shown to improve viral clearance in the treatment of chronic hepatitis C (210). In the treatment of sepsis and septic shock, a phase I clinical trial was completed in 2018. After enrolment of 38 patients, there were no unexpected safety findings, and no increase in pro-inflammatory cytokines (211).

As an anti-apoptotic cytokine, Interleukin 7 (IL-7) is necessary for clonal expansion and lymphocyte survival and induces the proliferation of CD4^+^ and CD8^+^ T cells. *Ex-vivo* experiments had shown that IL-7 increases T cell cytokine production and normalizes peripheral blood lymphocyte metabolism in patients with septic shock (212). Due to its key role in the development, maturation, expansion and homeostasis of B and T lymphocytes and its manifold effects on innate and adaptive immunity, IL-7 has been called the “maestro of the immune system” (213). Its efficacy in the treatment of viral infections has already been demonstrated in clinical studies with HIV patients (214). In combination with further *ex-vivo* results, the potential to restore important immunological defects in patients with sepsis could be demonstrated (215). In 2018, “IRIS-7” was published as a prospective, randomized, double-blind, placebo-controlled study in 27 patients with septic shock and severe lymphopenia (216). This study aimed for the first time at defects in adaptive immunity in the context of immunoadjuvant therapy. The use of recombinant human IL-7 increased the absolute lymphocyte count and the numbers of circulating CD4^+^ and CD8^+^ T cells, and there was no evidence for an increased pro-inflammatory response or a worsening of organ dysfunction.

In a recent *ex-vivo* study comparing patients with sepsis due to multi-resistant bacteria and critically ill but non-septic patients, septic patients showed increased expression of PD-1 and PD-L1 (217). Administration of both anti-PD-L1 and IL-7 resulted in increased IFN-γ production by T cells. Patients whose T cells could not be stimulated to increase production of IFN-γ showed a trend toward increased mortality.

The results obtained so far show that immunomodulation to restore and reorganize adaptive immunity may become a powerful tool for the future treatment of sepsis. However, further immunological phenotyping of critically ill patients with sepsis is needed to identify target groups. For this, robust diagnostic tools must be developed to identify this group of patients quickly and reliably (218). Also, meaningful prospective studies to validate biologically plausible hypotheses are still pending, as well as randomized controlled studies that show clinical benefit of these interventions (219).

#### Immunoglobulins

**Figure 4:**
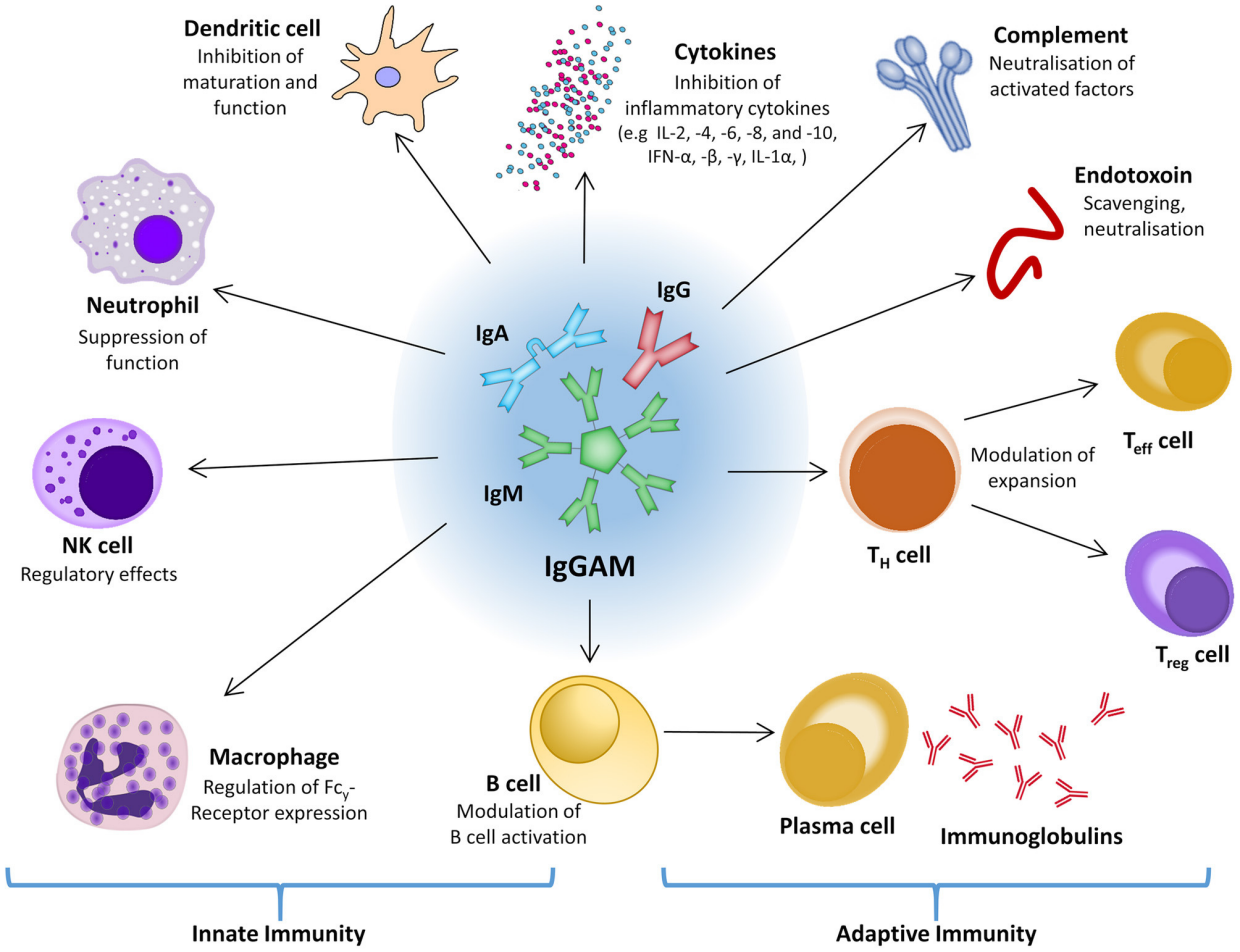
The central role of IgGAM in the innate and adaptive immune response. IFN, interferon; Ig, immunoglobulin; IgGAM, immunoglobulin G/A/M; IL, interleukin; NK cell, natural killer cell; T_eff_ cell, effector T cell; T_H_ cell, helper T cell; T_reg_ cell, regulatory T cell.

On the assumption that immune dysregulation and acquired immunosuppression are significant factors during sepsis and septic shock, stimulation of the immune response and/or substitution of individual immune system components might be a promising therapeutic approach. Within the usually well-balanced network of interacting and regulating factors of the immune system and the inflammatory response, polyvalent intravenous immunoglobulins (IVIG) might be an effective intervention to positively affect both pro- and anti-inflammatory processes (Figure 4) (220, 221). Experimental data show that polyvalent immunoglobulins can neutralize exo- and endotoxin, interact with complement factors, and improve pathogen phagocytosis by opsonization (222–225). For fulminant TSS, which is caused by staphylococcal or streptococcal superantigens, and for Kawasaki disease the use of adjunctive polyclonal IVIG preparations is recommended with moderate evidence (226–229).

In a *post-hoc* analysis of the CIGMA trial, which evaluated the efficacy of an IgM- and IgA-enriched polyclonal antibody preparation in patients with severe community-acquired pneumonia, a significant relative reduction in all-cause mortality of 54–68% was shown in a subgroup with high CRP, low IgM and a high CRP/low IgM ratio at baseline compared to placebo (230). Currently, the only available IgGAM preparation is Pentaglobin^®^, in which the content of IgM and IgA is enriched to 12% each. The formulation also contains neutralizing and toxin-binding antibodies against numerous Gram-positive and Gram-negative bacteria and modulates the effect of other pro-inflammatory (IFN-y, IL-6) as well as anti-inflammatory cytokines (IL-10) during lymphocyte response (225, 231, 232).

Although IVIG is widely used in the treatment of neurological, immunological and hematological diseases, the Surviving Sepsis Campaign (SSC) guidelines do not recommend the use of classical IVIG preparations containing almost exclusively IgG for sepsis and septic shock (145). In 2007, a randomized controlled multi-center study with 653 patients showed no survival benefit for the use of iv-immunoglobulin G (233). Even though generally well-tolerated, the administration of IVIG is not completely free of risks. In addition to side effects like hyperviscosity syndrome with thromboembolic events in some patients, cases of acute renal failure have been described, which are, however, presumably due to added stabilizers in the IVIG preparations (234).

In summary, the use of IVIG in the treatment of sepsis and septic shock remains controversial. Up to now, there is little reliable data due to highly variable study protocols, patient heterogeneity and inconsistency in the spectrum of analyzed laboratory parameters (235). It is expected, however, that ongoing RCTs (e.g., PEPPER-Trial; Personalized medicine with IgGAM compared with standard of care for treatment of peritonitis after source control) will provide more conclusive information (236).

#### Use of Artificial Intelligence in Sepsis, Gene Expression

Early identification of prognostic indicators from the vast amount of clinical and biochemical data is difficult and inconclusive. Survivors of sepsis often suffer from multiple long-term sequelae that can affect their quality of life and significantly shorten their life expectancy (237).

Current research is aimed at identifying biomarkers to help identify a possible severe clinical course at an early stage and to improve outcome through individualized therapy management. A landmark paper by Davenport et al. demonstrated in 2016 substantial heterogeneity in the individual host response to sepsis when investigating the transcriptome. At least two distinct sepsis response patterns (SRS1 and SRS2) could be identified with SRS1 being characterized by relative immunosuppression, endotoxin tolerance and metabolic derangement. These features were significantly associated with higher short-term mortality (238).

Using existing datasets of genetic expressions of septic patients, artificial intelligence (AI) systems are trained to recognize disease progression and clinical outcomes. In a recent publication, Banerjee et al. describe the use of a dataset of 228 pediatric patients with gene expression profiles collected within 24 h of ICU admission, through which an AI system was trained by the use of machine learning in multiple phases (239). In several steps, 20 differentiated expressed genes already associated with prediction of complicated course outcomes were identified. Based on further processing and training steps of this system, it was finally possible to identify 8 biomarkers that are known to be associated with an overshooting innate immune system. These biomarkers have previously been associated with sepsis mortality, now, however, show a predictive association with the severity of the disease course, even in surviving patients.

For example, matrix metalloproteinase 8 (MMP8) and resistin (RETN) have been identified to be associated with the release of TNF-α (240). Primarily expressed by macrophages and mononuclear cells, MMP8 itself shows beneficial anti-inflammatory activity in animal studies. MMP8 knockout mice show decreased NET activity, whereas in septic patients NET activation leads to NETosis, which in turn is positively correlated with severity (241, 242). This machine learning approach further identified lipocalin-2 (LCN2), which is known to be involved in microbiome homeostasis, in particular in protection of intestinal epithelia against oxidative stress. This immunosuppressive protein is considered a “hot candidate” for therapeutic use in abdominal sepsis (243, 244). Kangelaris et al. investigated genetic expression changes in septic patients with ARDS, and identified membrane metalloendopeptidase (MME) and hydroxycarboxylic acid receptor 3 (HCAR3) as candidates (245). Genes overexpressed in ARDS are frequently associated with poor outcome in sepsis, including MMP8 and RETN. Also, overexpression of MMP8, olfactomedin 4 (OLFM4), and interleukin 1 receptor type 2 (IL1R2) is associated with disease severity and the occurrence of organ failure in patients with AKI (246, 247).

In 2019, Seymour et al. published a paper describing the application of machine learning to readily available clinical data (rather than the genome or transcriptome analysis) (248). Data sets containing data from a total of 20,189 patients fulfilling sepsis-3 definition within 6 h of hospital admission were investigated. Based on the analysis of 29 sepsis-related variables (including demographics, vital signs, inflammation markers and markers of organ dysfunction), four distinct sepsis phenotypes (α, β, γ, and δ) could be differentiated. These phenotypes differed multidimensionally in terms of demographics, organ dysfunction, and laboratory values, but showed similarities of immune response, clinical outcome and response to therapy within the respective subclass. Using conventional analysis of sepsis subcategories such as site of infection, severity of illness, or organ dysfunction, these phenotypes cannot be captured. The early availability of the clinical parameters upon which this analysis was based may enable early identification of the respective phenotype and thus a potentially individualized therapy.

In summary, the approach using genome-wide association studies together with the application of AI using machine learning methods to available clinical data most probably may help to identify further markers and patient subclasses which are associated with severity and outcome. It is expected that suitable panels derived from clinical signs and peripheral blood samples will enable prognosis at an early stage and with little effort.

## Conclusion

Today, the cornerstones in therapy of sepsis and septic shock still consist of early focus control, timely administration of anti-infective drugs and hemodynamic stabilization through fluids and vasopressors. Over the last decades, a paradigm change is taking place, shifting the focus from the pathogen to the host when examining sepsis pathophysiology. Clinical understanding is continuously developing toward an immunological perspective. Complex pro- and anti-inflammatory pathways and disorders of the complement and coagulation system have been elucidated, thus revealing the heterogeneity and complexity of the syndrome. Alas, it has not yet been possible to transform knowledge into evidence-based practice for the effective treatment of sepsis.

Despite progress in the (further) development of innovative therapeutic approaches, such as targeted immune modulation, the use of novel anti-infective substances or methods for extracorporeal blood purification, there are still no effective adjunctive measures for which sufficient evidence has been provided. Since the search for drug-based sepsis therapies has proven unsuccessful in recent years, more focus should be placed on methodologically innovative approaches of research. The previous approach of using exclusively anti-inflammatory therapies has been disappointing, and the investigation of strategies aiming at re-balancing the profound immune dysregulation during sepsis and septic shock seems to be a promising goal.

## Author Contributions

All authors listed have made a substantial, direct, and intellectual contribution to the work and approved it for publication.

## Conflict of Interest

DJ has received lecture honoraria and travel reimbursement from ADVITOS and CytoSorbents Europe GmbH. SK received research support from Ambu, Daiichi Sankyo, ETView Ltd., Fisher & Paykel, Pfizer and Xenios. He also received lecture fees from Astra, C. R. Bard, Baxter, Biotest, Cytosorbents, Daiichi Sankyo, Fresenius, Gilead, Mitsubishi Tanabe Pharma, MSD, Pfizer, Philips, and Zoll. He received consultant fees from Bayer, Fresenius, Gilead, MSD and Pfizer. AN has received lecture honoraria and travel reimbursement from ThermoFisher Scientific GmbH, CytoSorbents Europe GmbH and Biotest AG, Germany over the past 5 years. The remaining author declares that the research was conducted in the absence of any commercial or financial relationships that could be construed as a potential conflict of interest.

## Abbreviations

ADAMTS-13: “a disintegrin and metalloproteinase with a thrombospondin type 1 motif, member 13”
AI: artificial intelligence
APC: antigen-presenting cell
CPFA: coupled plasma filtration adsorption
CRP: C-reactive protein
DAD: diffuse alveolar damage
DAMP: damage associated molecular pattern
DIC: disseminated intravascular coagulation
DNA: desoxyribonucleic acid
HLA-DR: human leukocyte antigen-D related
ICU: intensive care unit
IFN: interferon
IL: interleukin
Ig: immunoglobulin(s)
ISTH: International Society on Thrombosis and Haemostasis
IVIG: intravenous immunoglobulin
IgGAM: immunoglobulin G/A/M
KIM-1: kidney injury molecule-1
MBC: minimum bactericidal concentration
MDSC: myeloid-derived suppressor cell
MHC: major histocompatibility complex
MIC: minimum inhibitory concentration
mPTP: mitochondrial permeability transition pores
MR-proADM: mid-regional proadrenomedullin
mtDNA: mitochondrial DNA
NET: neutrophil extracellular trap
NF-κB: nuclear factor kappa-light-chain-enhancer of activated B cells
NGAL: neutrophil gelatinase-associated lipocalin
NK cell: natural killer cell
NO: nitric oxide
NOS: nitric oxide synthase
PAMP: pathogen-associated molecular pattern
PD-1: programmed death protein 1
PD1-R: programmed death protein 1-receptor
PICS, persistent inflammation: immunosuppression and catabolism syndrome
PMX: polymyxin B-immobilized fiber columns
PRR: pathogen recognition receptor
RCT: randomized controlled trial
RNS: reactive nitrogen species
ROS: reactive oxygen species
sa-AKI: sepsis associated acute kidney injury
SIC: sepsis-induced coagulopathy
TAMOF: thrombocythopenia-assiciated multiple-organ failure
TEC: tubular epithelial cell
T_eff_ cell: effector T cell
TF: tissue factor
TGF-ß: transforming growth factor-ß
T_H_ cell: helper T cell
TLR: toll-like receptor
TMA: thrombotic microangiopathy
TNF-α: tumor necrosis factor alpha
TPE: therapeutic plasma exchange
T_reg_ cell: regulatory T cell
TSS: toxic shock syndrome
vWF: von Willebrand factor
vWFCP: von Willebrand factor cleaving protease.
